# Supplementary material for: Tumorigenicity decrease in Bcl-xL deficient MDCK cells ensuring the safety for influenza vaccine production
Source: PLoS One. 2024 Dec 16;19(12):e0311069. doi: 10.1371/journal.pone.0311069 (PMC11649150; doi:10.1371/journal.pone.0311069)
Supplement: S1 File — (ZIP) [file pone.0311069.s002.zip › S2_File/4_KOG_enrichment/enrichmap/B_vs_A.up_KOG_enrichment_map.pdf]

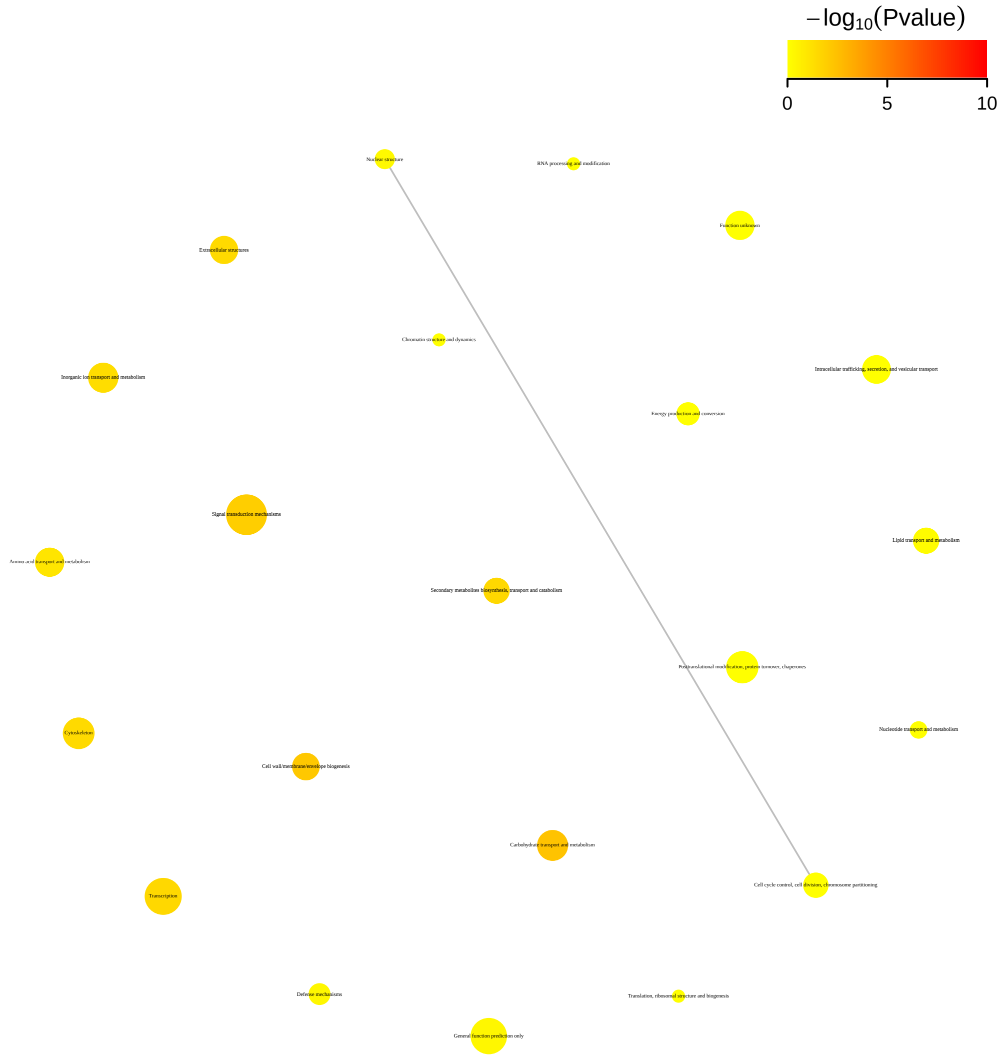

−log10(Pvalue)
0
5
10
Nuclear structure
RNA processing and modification
Function unknown
Extracellular structures
Chromatin structure and dynamics
Inorganic ion transport and metabolism
Intracellular trafficking, secretion, and vesicular transport
Energy production and conversion
Signal transduction mechanisms
Lipid transport and metabolism
Amino acid transport and metabolism
Secondary metabolites biosynthesis, transport and catabolism
Posttranslational modification, protein turnover, chaperones
Nucleotide transport and metabolism
Cytoskeleton
Cell wall/membrane/envelope biogenesis
Carbohydrate transport and metabolism
Cell cycle control, cell division, chromosome partitioning
Transcription
Defense mechanisms
Translation, ribosomal structure and biogenesis
General function prediction only
